# Supplementary material for: Investigating the HIV epidemic among Black gay and bisexual men in the Southern United States: Results of the HPTN 096 pilot cross-sectional assessment
Source: PLoS One. 2025 Oct 17;20(10):e0334031. doi: 10.1371/journal.pone.0334031 (PMC12533853; doi:10.1371/journal.pone.0334031)
Supplement: S1 Table — (DOCX) [file pone.0334031.s001.docx]

| **Pair** | **Study Community** | **Counties/ Parishes** | **Randomization** | **Population**  **(Black men, 2019)** | **HIV Prevalence (Black men, 2019, per 100,000)** | **HIV Viral Suppression**  **(Black MSM, 2019, %)** |
| --- | --- | --- | --- | --- | --- | --- |
| **Pilot Communities** | | | | | | |
| 1 | Dallas, TX | Dallas, Tarrant | Intervention | 354,985 | 1,676 | 55 |
|  | Houston, TX | Harris | Standard-of-Care | 332,027 | 1,745 | 50 |
| 2 | Montgomery, AL | Montgomery, Elmore, Autauga | Intervention | 59,554 | 1,553 | 66 |
|  | Greenville, SC | Greenville, Spartanburg | Standard-of-Care | 59,780 | 1,502 | 76 |
| **Non-Pilot Communities** | | | | | | |
| 3 | Memphis, TN | Shelby | Intervention | 182,276 | 1,934 | 65 |
|  | Charlotte, NC | Mecklenburg | Standard-of-Care | 128,228 | 2,228 | 69 |
| 4 | Atlanta, GA | Cobb, DeKalb, Fulton, Gwinnett | Intervention | 499,432 | 3,364 | 63 |
|  | Washington DC/  MD suburbs | Washington DC, Montgomery, Prince Georges County | Standard-of-Care | 408,194 | 2,827 | 62 |
| 5 | Birmingham, AL | Jefferson, Shelby, Tuscaloosa | Intervention | 139,262 | 1,504 | 67 |
|  | Columbia, SC | Lexington, Richland, Sumter | Standard-of-Care | 110,565 | 2,101 | 70 |
| 6 | Mobile, AL | Mobile, Baldwin, Jackson (MS) | Intervention | 72,651 | 1,430 | 57 |
|  | Charleston, SC | Charleston, Dorchester, Berkeley | Standard-of-Care | 76,752 | 1,501 | 69 |
| 7 | Ft. Lauderdale, FL | Broward | Intervention | 208,128 | 2,473 | 64 |
|  | Baton Rouge/  New Orleans, LA | East Baton Rouge, Orleans, Jefferson | Standard-of-Care | 203,184 | 2,504 | 68 |
| 8 | Orlando, FL | Orange | Intervention | 108,623 | 2,196 | 61 |
|  | Jackson, MS | Hinds, Madison, Rankin | Standard-of-Care | 90,405 | 1,866 | 53 |
