# Supplementary material for: Investigating the HIV epidemic among Black gay and bisexual men in the Southern United States: Results of the HPTN 096 pilot cross-sectional assessment
Source: PLoS One. 2025 Oct 17;20(10):e0334031. doi: 10.1371/journal.pone.0334031 (PMC12533853; doi:10.1371/journal.pone.0334031)
Supplement: Attachment — Submitted filename: Response to Reviewers.docx [file pone.0334031.s003.docx]

To: Douglas S. Krakower, MD, Academic Editor, PLOS ONE

Dear Dr. Krakower,

Thank you for the opportunity to move forward with our manuscript submission. We have addressed all reviewer feedback, as outlined in the appendix to this letter. In addition, we have revised our financial statement, competing interests and data availability statements, as shown below.

**Revised Funding Statement:** Overall support for the HIV Prevention Trials Network (HPTN) is provided by the National Institute of Allergy and Infectious Diseases (NIAID), Office of the Director (OD), National Institutes of Health (NIH), National Institute on Drug Abuse (NIDA), the National Institute of Mental Health (NIMH), and the Eunice Kennedy Shriver National Institute of Child Health and Human Development (NICHD) under Award Numbers UM1AI068619 (HPTN Leadership and Operations Center), UM1AI068617 (HPTN Statistical and Data Management Center), and UM1AI068613 (HPTN Laboratory Center). Neither D3 Consulting LLC nor Equity Solutions LLC, both which are single-member consulting companies, provided any funding for the study. There was no additional external funding received for this study.

**Revised Competing Interests Statement:** Per the journal’s policy, some authors have competing interests. Both DRB and MJS served as representatives of the funder, NIAID and NIMH, respectively, and both played roles in the study design, data analysis and interpretation, decision to publish, and preparation of the manuscript. Both DDD and CHO received funding from NIH through the award identified in the funding statement for their participation in study design, data analysis and interpretation, and preparation of the manuscript; however, their individual LLCs (D3 Consulting and Equity Solutions) did not play any role in these activities. These competing interests do not alter our adherence to PLOS ONE policies on sharing data and materials.

**Revised Data Availability Statement:** The data supporting the findings of this study are available at the Harvard Dataverse at <https://dataverse.harvard.edu/dataverse/HPTN096/>.

We are submitting the revised manuscript with both a version with tracked changes, and version with all of the revisions accepted.

Please let us know if there is additional feedback or questions.

With kind regards,

Chris Beyrer, MD

**Appendix: Response to Reviewer Comments**

1. Please ensure that your manuscript meets PLOS ONE's style requirements, including those for file naming. The PLOS ONE style templates can be found at

[https://journals.plos.org/plosone/s/file?id=wjVg/PLOSOne_formatting_sample_main_body.pdf](https://urldefense.com/v3/__https:/journals.plos.org/plosone/s/file?id=wjVg*PLOSOne_formatting_sample_main_body.pdf__;Lw!!ELf_LxN3sEQ!YLgfwigkm6-LeiSdprXq1Hc_wRim5iwuLXe7auNXWXZj_07CbkqdcJwbNGUmmbr2uhJSP6yFYwR75PnrJrtVZOVyeg$) and [https://journals.plos.org/plosone/s/file?id=ba62/PLOSOne_formatting_sample_title_authors_affiliations.pdf](https://urldefense.com/v3/__https:/track.editorialmanager.com/CL0/https:*2F*2Fjournals.plos.org*2Fplosone*2Fs*2Ffile*3Fid=ba62*2FPLOSOne_formatting_sample_title_authors_affiliations.pdf/1/010f01982c690517-77d93bc4-d111-4602-999a-4549f260b263-000000/BXuhlXFlFfnpzXIx5SAnjWYIZWYNBgBNaVyS9e_SleA=220__;JSUlJSUlJQ!!ELf_LxN3sEQ!YLgfwigkm6-LeiSdprXq1Hc_wRim5iwuLXe7auNXWXZj_07CbkqdcJwbNGUmmbr2uhJSP6yFYwR75PnrJrtgr4_9RA$)

**Response:** We have reviewed the formatting guidelines for both the main body and cover page of the manuscript and completed all of the revisions.

______________________________________________________________________________

2. Thank you for stating in your Funding Statement:

[Overall support for the HIV Prevention Trials Network (HPTN) is provided by the National Institute of Allergy and Infectious Diseases (NIAID), Office of the Director (OD), National Institutes of Health (NIH), National Institute on Drug Abuse (NIDA), the National Institute of Mental Health (NIMH), and the Eunice Kennedy Shriver National Institute of Child Health and Human Development (NICHD) under Award Numbers UM1AI068619 (HPTN Leadership and Operations Center), UM1AI068617 (HPTN Statistical and Data Management Center), and UM1AI068613 (HPTN Laboratory Center].

Please provide an amended statement that declares *all* the funding or sources of support (whether external or internal to your organization) received during this study, as detailed online in our guide for authors at [http://journals.plos.org/plosone/s/submit-now](https://urldefense.com/v3/__http:/journals.plos.org/plosone/s/submit-now__;!!ELf_LxN3sEQ!YLgfwigkm6-LeiSdprXq1Hc_wRim5iwuLXe7auNXWXZj_07CbkqdcJwbNGUmmbr2uhJSP6yFYwR75PnrJruuGSJ0sQ$).  Please also include the statement “There was no additional external funding received for this study.” in your updated Funding Statement.

Please include your amended Funding Statement within your cover letter. We will change the online submission form on your behalf.

**Response:** The revised Funding Statement is included in the main body of this letter.

______________________________________________________________________________

3. Thank you for stating the following in your manuscript:

[Overall support for the HIV Prevention Trials Network (HPTN) is provided by the National Institute of Allergy and Infectious Diseases (NIAID), Office of the Director (OD), National Institutes of Health (NIH), National Institute on Drug Abuse (NIDA), the National Institute of Mental Health (NIMH), and the Eunice Kennedy Shriver National Institute of Child Health and Human Development (NICHD) under Award Numbers UM1AI068619 (HPTN Leadership and Operations Center), UM1AI068617 (HPTN Statistical and Data Management Center), and UM1AI068613 (HPTN Laboratory Center).]

We note that you have provided funding information that is currently declared in your Funding Statement. However, funding information should not appear in the Acknowledgments section or other areas of your manuscript. We will only publish funding information present in the Funding Statement section of the online submission form.

Please remove any funding-related text from the manuscript and let us know how you would like to update your Funding Statement. Currently, your Funding Statement reads as follows:

[Overall support for the HIV Prevention Trials Network (HPTN) is provided by the National Institute of Allergy and Infectious Diseases (NIAID), Office of the Director (OD), National Institutes of Health (NIH), National Institute on Drug Abuse (NIDA), the National Institute of Mental Health (NIMH), and the Eunice Kennedy Shriver National Institute of Child Health and Human Development (NICHD) under Award Numbers UM1AI068619 (HPTN Leadership and Operations Center), UM1AI068617 (HPTN Statistical and Data Management Center), and UM1AI068613 (HPTN Laboratory Center]

Please include your amended statements within your cover letter; we will change the online submission form on your behalf.

**Response:** The revised Funding Statement is included in the main body of this letter. In addition, all funding-related information has been removed from the main body of the manuscript.

______________________________________________________________________________

4. When completing the data availability statement of the submission form, you indicated that you will make your data available on acceptance. We strongly recommend all authors decide on a data sharing plan before acceptance, as the process can be lengthy and hold up publication timelines. Please note that, though access restrictions are acceptable now, your entire data will need to be made freely accessible if your manuscript is accepted for publication. This policy applies to all data except where public deposition would breach compliance with the protocol approved by your research ethics board. If you are unable to adhere to our open data policy, please kindly revise your statement to explain your reasoning and we will seek the editor's input on an exemption. Please be assured that, once you have provided your new statement, the assessment of your exemption will not hold up the peer review process.

**Response:** The data supporting this manuscript has been made to the public via, and can be found here: <https://dataverse.harvard.edu/dataverse/HPTN096/>, and the data availability statement is below.

**Data availability statement:** The data supporting the findings of this study are available at the Harvard Dataverse at <https://dataverse.harvard.edu/dataverse/HPTN096/>. ______________________________________________________________________________

5a. Thank you for stating the following in the Competing Interests section:

[The authors have declared that no competing interests exist.].

We note that one or more of the authors are employed by a commercial company: D3 Consulting LLC

Please provide an amended Funding Statement declaring this commercial affiliation, as well as a statement regarding the Role of Funders in your study. If the funding organization did not play a role in the study design, data collection and analysis, decision to publish, or preparation of the manuscript and only provided financial support in the form of authors' salaries and/or research materials, please review your statements relating to the author contributions, and ensure you have specifically and accurately indicated the role(s) that these authors had in your study. You can update author roles in the Author Contributions section of the online submission form.

Please also include the following statement within your amended Funding Statement.

“The funder provided support in the form of salaries for authors [insert relevant initials], but did not have any additional role in the study design, data collection and analysis, decision to publish, or preparation of the manuscript. The specific roles of these authors are articulated in the ‘author contributions’ section.”

If your commercial affiliation did play a role in your study, please state and explain this role within your updated Funding Statement.

**Response:** The revised Funding Statement and Competing Interests section is included in the main body of this letter.

______________________________________________________________________________

5b. Please also provide an updated Competing Interests Statement declaring this commercial affiliation along with any other relevant declarations relating to employment, consultancy, patents, products in development, or marketed products, etc.

Within your Competing Interests Statement, please confirm that this commercial affiliation does not alter your adherence to all PLOS ONE policies on sharing data and materials by including the following statement: "This does not alter our adherence to  PLOS ONE policies on sharing data and materials.” (as detailed online in our guide for authors [http://journals.plos.org/plosone/s/competing-interests](https://urldefense.com/v3/__http:/journals.plos.org/plosone/s/competing-interests__;!!ELf_LxN3sEQ!YLgfwigkm6-LeiSdprXq1Hc_wRim5iwuLXe7auNXWXZj_07CbkqdcJwbNGUmmbr2uhJSP6yFYwR75PnrJrsJhiJmkQ$)) . If this adherence statement is not accurate and  there are restrictions on sharing of data and/or materials, please state these. Please note that we cannot proceed with consideration of your article until this information has been declared.

Please include both an updated Funding Statement and Competing Interests Statement in your cover letter. We will change the online submission form on your behalf.

**Response:** The revised Funding Statement and Competing Interests section is included in the main body of this letter.

______________________________________________________________________________

6. We note that Figure 4 in the Supplementary File of your submission contains map image which may be copyrighted. All PLOS content is published under the Creative Commons Attribution License (CC BY 4.0), which means that the manuscript, images, and Supporting Information files will be freely available online, and any third party is permitted to access, download, copy, distribute, and use these materials in any way, even commercially, with proper attribution. For these reasons, we cannot publish previously copyrighted maps or satellite images created using proprietary data, such as Google software (Google Maps, Street View, and Earth). For more information, see our copyright guidelines: [http://journals.plos.org/plosone/s/licenses-and-copyright](https://urldefense.com/v3/__http:/journals.plos.org/plosone/s/licenses-and-copyright__;!!ELf_LxN3sEQ!YLgfwigkm6-LeiSdprXq1Hc_wRim5iwuLXe7auNXWXZj_07CbkqdcJwbNGUmmbr2uhJSP6yFYwR75PnrJrtGn4j-WQ$).

We require you to either (1) present written permission from the copyright holder to publish these figures specifically under the CC BY 4.0 license, or (2) remove the figures from your submission:

1. You may seek permission from the original copyright holder of Figure 4 in your Supplementary File to publish the content specifically under the CC BY 4.0 license.

We recommend that you contact the original copyright holder with the Content Permission Form ([http://journals.plos.org/plosone/s/file?id=7c09/content-permission-form.pdf](https://urldefense.com/v3/__http:/journals.plos.org/plosone/s/file?id=7c09*content-permission-form.pdf__;Lw!!ELf_LxN3sEQ!YLgfwigkm6-LeiSdprXq1Hc_wRim5iwuLXe7auNXWXZj_07CbkqdcJwbNGUmmbr2uhJSP6yFYwR75PnrJrsmns9Twg$)) and the following text:

“I request permission for the open-access journal PLOS ONE to publish XXX under the Creative Commons Attribution License (CCAL) CC BY 4.0 ([http://creativecommons.org/licenses/by/4.0/](https://urldefense.com/v3/__http:/creativecommons.org/licenses/by/4.0/__;!!ELf_LxN3sEQ!YLgfwigkm6-LeiSdprXq1Hc_wRim5iwuLXe7auNXWXZj_07CbkqdcJwbNGUmmbr2uhJSP6yFYwR75PnrJrvs9uBG2Q$)). Please be aware that this license allows unrestricted use and distribution, even commercially, by third parties. Please reply and provide explicit written permission to publish XXX under a CC BY license and complete the attached form.”

Please upload the completed Content Permission Form or other proof of granted permissions as an "Other" file with your submission.

In the figure caption of the copyrighted figure, please include the following text: “Reprinted from [ref] under a CC BY license, with permission from [name of publisher], original copyright [original copyright year].”

2. If you are unable to obtain permission from the original copyright holder to publish these figures under the CC BY 4.0 license or if the copyright holder’s requirements are incompatible with the CC BY 4.0 license, please either i) remove the figure or ii) supply a replacement figure that complies with the CC BY 4.0 license. Please check copyright information on all replacement figures and update the figure caption with source information. If applicable, please specify in the figure caption text when a figure is similar but not identical to the original image and is therefore for illustrative purposes only.

The following resources for replacing copyrighted map figures may be helpful:

USGS National Map Viewer (public domain): [http://viewer.nationalmap.gov/viewer/](https://urldefense.com/v3/__http:/viewer.nationalmap.gov/viewer/__;!!ELf_LxN3sEQ!YLgfwigkm6-LeiSdprXq1Hc_wRim5iwuLXe7auNXWXZj_07CbkqdcJwbNGUmmbr2uhJSP6yFYwR75PnrJrvaR7GB6g$)
The Gateway to Astronaut Photography of Earth (public domain): [http://eol.jsc.nasa.gov/sseop/clickmap/](https://urldefense.com/v3/__http:/eol.jsc.nasa.gov/sseop/clickmap/__;!!ELf_LxN3sEQ!YLgfwigkm6-LeiSdprXq1Hc_wRim5iwuLXe7auNXWXZj_07CbkqdcJwbNGUmmbr2uhJSP6yFYwR75PnrJrvjN5YlSA$)
Maps at the CIA (public domain): [https://www.cia.gov/library/publications/the-world-factbook/index.html](https://urldefense.com/v3/__https:/www.cia.gov/library/publications/the-world-factbook/index.html__;!!ELf_LxN3sEQ!YLgfwigkm6-LeiSdprXq1Hc_wRim5iwuLXe7auNXWXZj_07CbkqdcJwbNGUmmbr2uhJSP6yFYwR75PnrJrvsJ6oijQ$) and [https://www.cia.gov/library/publications/cia-maps-publications/index.html](https://urldefense.com/v3/__https:/track.editorialmanager.com/CL0/https:*2F*2Fwww.cia.gov*2Flibrary*2Fpublications*2Fcia-maps-publications*2Findex.html/1/010f01982c690517-77d93bc4-d111-4602-999a-4549f260b263-000000/vYWPAIP9YeUBGKcSkueLc2kXgb0yAv1rRd9-y_GhCgs=220__;JSUlJSUl!!ELf_LxN3sEQ!YLgfwigkm6-LeiSdprXq1Hc_wRim5iwuLXe7auNXWXZj_07CbkqdcJwbNGUmmbr2uhJSP6yFYwR75PnrJrvH-QNuNg$)
NASA Earth Observatory (public domain): [http://earthobservatory.nasa.gov/](https://urldefense.com/v3/__http:/earthobservatory.nasa.gov/__;!!ELf_LxN3sEQ!YLgfwigkm6-LeiSdprXq1Hc_wRim5iwuLXe7auNXWXZj_07CbkqdcJwbNGUmmbr2uhJSP6yFYwR75PnrJrvck6Db8A$)
Landsat: [http://landsat.visibleearth.nasa.gov/](https://urldefense.com/v3/__http:/landsat.visibleearth.nasa.gov/__;!!ELf_LxN3sEQ!YLgfwigkm6-LeiSdprXq1Hc_wRim5iwuLXe7auNXWXZj_07CbkqdcJwbNGUmmbr2uhJSP6yFYwR75PnrJrsdLZ7XSA$)
USGS EROS (Earth Resources Observatory and Science (EROS) Center) (public domain): [http://eros.usgs.gov/#](https://urldefense.com/v3/__http:/eros.usgs.gov/*__;Iw!!ELf_LxN3sEQ!YLgfwigkm6-LeiSdprXq1Hc_wRim5iwuLXe7auNXWXZj_07CbkqdcJwbNGUmmbr2uhJSP6yFYwR75PnrJrs6xhdyAg$)
Natural Earth (public domain): [http://www.naturalearthdata.com/](https://urldefense.com/v3/__http:/www.naturalearthdata.com/__;!!ELf_LxN3sEQ!YLgfwigkm6-LeiSdprXq1Hc_wRim5iwuLXe7auNXWXZj_07CbkqdcJwbNGUmmbr2uhJSP6yFYwR75PnrJrvCoiY0Eg$)

**Response:** We have included only one supplemental file, and it is a table, not an image. In addition, we don’t have any map images affiliated with the manuscript. The figures that are included in the main manuscript were generated by the authors using Excel.

______________________________________________________________________________

7. We notice that your supplementary tables are included in the manuscript file. Please remove them and upload them with the file type 'Supporting Information'. Please ensure that each Supporting Information file has a legend listed in the manuscript after the references list.

**Response:** We are now including the supplemental table as a separate file with the correct file name.

______________________________________________________________________________

8. Please include captions for your Supporting Information files at the end of your manuscript, and update any in-text citations to match accordingly. Please see our Supporting Information guidelines for more information: [http://journals.plos.org/plosone/s/supporting-information](https://urldefense.com/v3/__http:/journals.plos.org/plosone/s/supporting-information__;!!ELf_LxN3sEQ!YLgfwigkm6-LeiSdprXq1Hc_wRim5iwuLXe7auNXWXZj_07CbkqdcJwbNGUmmbr2uhJSP6yFYwR75PnrJrt4mqTR0A$).

If the reviewer comments include a recommendation to cite specific previously published works, please review and evaluate these publications to determine whether they are relevant and should be cited. There is no requirement to cite these works unless the editor has indicated otherwise.

**Response:** A Supporting Information section has been added at the end of the manuscript, along with the title for the one supplemental table.

______________________________________________________________________________

9. Please review your reference list to ensure that it is complete and correct. If you have cited papers that have been retracted, please include the rationale for doing so in the manuscript text, or remove these references and replace them with relevant current references. Any changes to the reference list should be mentioned in the rebuttal letter that accompanies your revised manuscript. If you need to cite a retracted article, indicate the article’s retracted status in the References list and also include a citation and full reference for the retraction notice.

**Response:** The reference list has been reviewed and revised.

______________________________________________________________________________

Review Comments to the Author

Please use the space provided to explain your answers to the questions above. You may also include additional comments for the author, including concerns about dual publication, research ethics, or publication ethics. (Please upload your review as an attachment if it exceeds 20,000 characters)

Reviewer #1: In this manuscript the Authors describe their findings from a cross-sectional assessment during the pilot phase of the HPTN 096 study. Overall, they found a very high burden of HIV in Black MSM at the study sites. Of those living with HIV, 77% were in HIV care. At two study sites, viral loads were obtained and only 70% were virally suppressed. Of those not living with HIV, only 22% reported being on PrEP, and of those, 12% had no evidence of PrEP on dried blood spots. Overall, the study Authors found a high burden of HIV in Black MSM and low rates of viral suppression and PrEP use in those not living with HIV.

I found this manuscript interesting and well-written and only have some minor suggestions:

1. "southern" should be capitalized throughout the manuscript when referring to "Southern United States".

**Response:** The word “southern” has been capitalized appropriately throughout the document.

______________________________________________________________________________

2. In the Introduction, both present and past tense are used. I would stick with past tense whenever applicable.

**Response:** This section was reviewed and revised to use the proper verb tense consistently.

______________________________________________________________________________

3. Line 251-253: This is in track change.

**Response:** This has been corrected.

______________________________________________________________________________

4. Line 276: This is in track change.

**Response:** This has been corrected.

______________________________________________________________________________

5. Line 285-286: What do you mean by "HIV testing was performed for the remaining four participants..." Please consider rephrasing.

**Response:** This has been reviewed and revised.

______________________________________________________________________________

6. Lines 411-414: Of those reporting taking PrEP but without detectable TFV-DP on their DBS, were you able to confirm that they were not on injectable PrEP (Cabotegravir was approved by the FDA for PrEP in 2021 before this study)?

**Response:** There was very little CAB-LA available to the study population (Black MSM) in the southern United States at the time of data collection (May through November 2022). However, we did ask participants if they had taken injectable long-acting cabotegravir, and everyone responded no; therefore, no testing was done for it.

______________________________________________________________________________

Reviewer #2: This is a remarkably well-written and comprehensive description of the cross-sectional study data from HPTN 096. I only have two minor points of clarification.

Were any efforts made to correct for the RDS component of the sampling strategy in the analysis?

**Response:** We did not use an RDS adjustment as we were not using the tool for population prevalence estimation, but rather as a recruitment enhancement when venues were too few for venue-time sampling.  This is the starfish sampling advantage for recruitment.

______________________________________________________________________________

Only 29 samples were tested for HIV drug resistance. A little more information about this and if there is any suspected bias in which participants had samples tested for resistance compared to those who did not would be helpful. Was this just due to the differences in lab accessibility across communities? It seems as though there are still missing samples even with this limitation.

**Response:** Only samples from individuals with HIV viral loads >500 copies/mL were tested for HIV drug resistance, as resistance testing requires a minimal level of virus. On 29 participants had samples that met this criteria, as defined in the methods section (HIV drug resistance testing).

______________________________________________________________________________

[NOTE: If reviewer comments were submitted as an attachment file, they will be attached to this email and accessible via the submission site. Please log into your account, locate the manuscript record, and check for the action link "View Attachments". If this link does not appear, there are no attachment files.]

While revising your submission, please upload your figure files to the Preflight Analysis and Conversion Engine (PACE) digital diagnostic tool, [https://pacev2.apexcovantage.com/](https://urldefense.com/v3/__https:/track.editorialmanager.com/CL0/https:*2F*2Fpacev2.apexcovantage.com*2F/1/010f01982c690517-77d93bc4-d111-4602-999a-4549f260b263-000000/XpU-tGbagry_6c895GQTGEiaMruJHi7bCKXGWmjWOyI=220__;JSUl!!ELf_LxN3sEQ!YLgfwigkm6-LeiSdprXq1Hc_wRim5iwuLXe7auNXWXZj_07CbkqdcJwbNGUmmbr2uhJSP6yFYwR75PnrJrtURhWuSw$). PACE helps ensure that figures meet PLOS requirements. To use PACE, you must first register as a user. Registration is free. Then, login and navigate to the UPLOAD tab, where you will find detailed instructions on how to use the tool. If you encounter any issues or have any questions when using PACE, please email PLOS at [figures@plos.org](mailto:figures@plos.org). Please note that Supporting Information files do not need this step.
